# Retraction Note: Impact of daily high dose oral vitamin D therapy on the inflammatory markers in patients with COVID 19 disease

**DOI:** 10.1038/s41598-022-10830-8

**Published:** 2022-04-20

**Authors:** Maheshwar Lakkireddy, Srikanth Goud Gadiga, R. D. Malathi, Madhu Latha Karra, I. S. S. V. Prasad Murthy Raju, Ragini, Sangeetha Chinapaka, K. S. S. Sai Baba, Manohar Kandakatla

**Affiliations:** 1 Department of Orthopaedics/Biochemistry/Internal Medicine, Nizam’s Institute of Medical Sciences, Punjagutta, Hyderabad, Telangana India; 2 Department of General Medicine/Biochemistry, Gandhi Medical College, Secunderabad, Telangana India

Retraction of: *Scientific Reports* 10.1038/s41598-021-90189-4, published online 20 May 2021

The Editors have retracted this Article.

After publication concerns were raised about several aspects of the study, in particular that at baseline there are large differences in the parameters measured indicating that randomisation may not have been performed correctly. Post-publication peer review has confirmed that the alternative allocation method used in this study is not appropriate for randomised clinical trials. This means that the patients were not correctly randomised and therefore the differences in outcome seen between the two arms of the study cannot be attributed to the Pulse D therapy. The Editors therefore no longer have confidence in the conclusions of this study.

Maheshwar Lakkireddy, R. D. Malathi, Madhu Latha Karra, Ragini, Sangeetha Chinapaka, and K. S. S. Sai Baba disagree with this retraction. Srikanth Goud Gadiga, I. S. S. V. Prasad Murthy Raju, and Manohar Kandakatla have not responded to correspondence from the Editors about this retraction.

